# Supplementary material for: Are carbon emissions trading and green financial instruments synergistic? -Comprehensive quantitative research based on content analysis
Source: PLoS One. 2024 Mar 7;19(3):e0298601. doi: 10.1371/journal.pone.0298601 (PMC10919601; doi:10.1371/journal.pone.0298601)
Supplement: S1 Data — (ZIP) [file pone.0298601.s001.zip › Data/地级市Z_F工作报告.zip!/╝¬┴╓/░╫│╟╩╨/└┤╫╘╣1⁄2╓┌║┼í╛┬φ┐╦╩2╛▌═°í┐_2010.docx]

各位代表：

　　现在，我代表白城市人民政府，向大会作政府工作报告，请予审议，并请市政协各位委员提出意见。

　　一、2009年工作回顾总结

　　2009年，是我们面对金融危机砥砺奋进、经受严峻考验的一年。在市委的正确领导下，在市人大、市政协的监督和支持下，紧紧依靠和带领全市人民，按照“保增长、增投资、促和谐”的工作布局，强力推进吉林西部重要的能源基地、粮食生产基地、特色农产品加工基地、区域商品集散地和生态产业基地“五大基地”建设，统筹发展社会事业，完成了市四届人大二次会议确定的各项任务，加快白城发展、实现崛起振兴迈出了坚实步伐。

　　——地区生产总值快速增长。预计全市地区生产总值实现355亿元，同比增长17.5%，增速比全省平均水平高出4个百分点。

　　——农业克服严重旱灾取得较好收成。全市粮食产量达到50.3亿斤，是历史上第二个高产年份。农业总产值实现125亿元，同比增长6.7%。

　　——工业经济企稳回升逆势上扬。全市规模以上工业实现增加值50亿元，同比增长18%，增幅高于全省平均水平2个百分点。

　　——商贸服务业继续保持繁荣活跃。全市实现社会消费品零售总额136亿元，同比增长19%。

　　——固定资产投资增势强劲。全市固定资产投资完成350亿元，同比增长33.5%，增速高于全省平均水平4.9个百分点。

　　——财政收入抵消不利影响逐步走高。全市地方级财政收入完成12.6亿元，同比增长15.8%。

　　——人民生活水平稳中提高。城镇居民人均可支配收入14800元。农村居民人均纯收入3490元。

　　——“发展杯”竞赛活动开展扎实有效。在2009年省政府考核评比2008年全省“发展杯”竞赛活动中，我市在全省9个市州中被省政府评为一等奖。这是近年来省政府综合性奖项中，我市获得的最好成绩。

　　这些成就，标志着我们综合实力不断增强、后发优势不断彰显，发展思路更加清晰、发展成效更加显著，必将增强全市人民负重拼搏、加快发展的信心，激励我们在新一轮竞相发展、科学发展中奋力前行。

　　一年来，我们主要做了以下十个方面工作：

　　（一）抢抓扩大内需政策机遇，强力推进重大项目建设。抓住国家实施一揽子投资计划契机，积极向上争取重大投资项目。全市共争取国家和省投资项目483个，资金近11亿元，超过全省平均水平，实现了历史性突破。加快推进千万元以上在建项目100个、新开项目100个、前期工作项目100个建设。现已滚动发展到335个，完成投资298亿元。全市亿元以上重大项目182个，是2008年的2倍多，投资额度、增速幅度均达到历史最好水平。特别是长白快速铁路、白城站扩能改造、白阿铁路线增建复线、嫩通高速公路、市区金辉公铁立交桥、白城民航机场等一批对白城长远发展具有战略性、支撑性的重大项目取得历史性进展。

　　（二）加大能源利用开发力度，抓好能源基地建设。中电投白城发电公司一期工程2×66万千瓦项目已投资35.2亿元，现已完成设备安装调试，今年6月并网发电。投资18.2亿元的白城热电厂供热改造项目启动建设。大安大唐、镇赉华能火电项目已完成了初可研。着眼建设千万千瓦级风电基地，整合全市风电资源，实行“五个统一”管理，推进风能资源开发规模化、产业化发展。目前，全市风电并网装机容量增至148万千瓦，占全国并网装机总量的10%左右。风电设备制造企业已发展到12户。白城500千伏输变电工程已建成运行。通榆500千伏输变电工程项目开工建设。规划实施了油气开发和光伏发电示范项目，大安油田、镇赉天然气开发项目积极推进。洮南、通榆金太阳示范工程得到了国家批准建设，全市多种能源并举的发展格局基本形成。

　　（三）推进粮食增产工程建设，壮大特色农产品加工业。土地开发整理、标准粮田建设、引嫩入白、大安灌区等11个工程、22个项目建设取得了阶段性成果。镇赉、大安生态农业示范区项目启动建设。实施百万亩玉米保护性耕作示范推广工程，保护性耕作推广面积已发展到25.7万亩。加快培育壮大产业化龙头企业，投资8亿元的福建东佳百万吨谷物粮油深加工、投资6.7亿元的镇赉金鹤乳业制品、投资3.3亿元的洮南6万吨花生油及蛋白加工等一批重点特色农畜产品加工项目开工建设。扶持壮大盛吉蓖麻加工、大安安大牧业、洮北龙丹乳业等重点龙头企业。全市农产品加工企业已发展到1065户，省级农业产业化重点龙头企业发展39户，市级农业产业化重点龙头企业发展48户。加强农业科技园区和庭院经济建设，全市新启动建设农业科技园区23个，占年初计划的115%，形成了集约发展效应。

　　（四）抓住企稳向好有利契机，加快工业经济发展步伐。充分发挥工业园区引领带动作用，白城工业园区入园项目已达到100个，超亿元项目24个，投产项目44个。白城经济开发区、民营经济开发区、查干浩特旅游经济开发区、大安经济开发区也都引进一批大项目、好项目。推进白城中一精锻连杆精锻件扩能改造、金事达40万套柴油发动机电控线束等20个较大技改项目建设。金升镍业、尼特固汽配等近50个工业项目实现投达产。全市规模以上工业企业增加到279户，占全年任务目标的107.3%。单位GDP能耗下降5.3%，规模以上工业万元增加值能耗下降7%。市直麻纺、盛华、中牧等8户企业已基本完成了招商重组，金鹏、红钻、通业等5户企业进入招商重组实质操作阶段。民营经济得到了发展壮大，预计全市民营经济主营业务收入完成322亿元，同比增长23.2%。

　　（五）实施吉林西部荒漠化治理工程，推进生态产业建设。统筹规划投资120亿元的吉林西部荒漠化治理工程，启动向海、莫莫格、嫩江湾湿地保护开发等37个项目。围绕大力发展绿色经济，组织开展“举市三年大造林、加快治理荒漠化”竞赛活动，秋备春造工程成效显著。规划启动百万亩苇田工程。实施200万亩盐碱地脱硫废渣改造项目一期工程，全市水田试验面积扩大到200亩、旱田试验面积600亩，均取得了较好的生态效益和经济效益。

　　（六）强化开发开放大通道建设，构建立体交通运输网络。珲乌高速公路白城段工程全面开工建设。完成了跨越吉林、黑龙江、内蒙古3省区，贯穿镇赉、洮南、通榆、洮北4个县（市、区）的嫩通高速公路前期工作。长白快速铁路、白阿铁路线增建复线、白城站扩能改造、市区金辉公铁立交桥等项目取得了积极进展。白城民航机场选址已获国家批准，完成了预可研，将使我市形成公路、铁路、航空立体对外通道。

　　（七）加大“走出去、请进来”力度，深入开展招商引资。利用国家“两会”契机，成功在北京举办了“白城（北京）招商引资项目签约仪式”。在长春举办外埠商会联谊会，与上海、广东等全国九大商会签署战略合作框架协议，实现了我市与外省商会战略合作的良好开局。在温州、宁波等一线、二线城市举办招商引资推介会、签约会。全市引进到位资金252亿元，同比增长26%。实际利用外资6396万美元，同比增长122.3%，增速位居全省第1位。积极搭建融资平台，建立起了以白城市国有资产经营投资有限公司为主的“一主四辅”融资平台。在长春成功举行了金融支持白城经济发展战略合作签约仪式，我市从省内4家金融机构获得3年400亿元授信额度，为项目建设、企业发展提供了资金支撑。

　　（八）加速县域经济发展，扎实推进社会主义新农村建设。深入实施县域突破战略，县（市）预计地区生产总值增长18%，地方级财政收入增长13.8%，规模以上工业增加值增长19.3%。地区生产总值、规模以上工业增加值增速高于全市平均水平，县域经济发展势头良好。县（市）工业集中区加快发展，基础设施建设、重大项目引进实现了新突破。启动小城镇建设，我市有11个小城镇入选全省“百镇建设工程”试点镇，已有8个试点镇开始建设。市级9个试点镇，已有2个试点镇开始建设。稳步推进新农村建设，全市新启动省、市、县、乡四级新农村建设推进村100个，农村面貌发生了可喜变化。

　　（九）努力保障和改善民生，有效推进和谐白城建设。尽全力办好民生民利实事。城镇新增就业4.5万人。城镇登记失业率控制在3.9%以内。开发公益性岗位保持在1.1万人的就业规模。新型农村合作医疗参合率达到87.1%。城镇居民基本医疗保险参保47.4万人。城镇职工基本医疗保险参保33.5万人。全部免除城乡义务教育阶段21.4万名学生学杂费。改造了151万平方米的棚户区及危旧房。建设城市廉租住房20万平方米。改造农村泥草房5.3万户。完成50个整村扶贫开发推进工作目标，实现脱贫人口3.2万人。解决2.8万人的饮水安全问题。建成150个农家书屋，250个农村文化大院。新建市、县、乡计生技术服务站8个。全面完成广播电视“村村通”和有线电视数字化整体转换工程。实施了采暖费补贴改革。提高了机关干部及事业单位职工阳光津贴标准。城市低保对象月人均补助标准提高到170元；农村低保对象年人均补助标准提高到760元。开展了整治城区环境、提升城市管理水平攻坚战。实施了长庆南街道路修建、幸福南大街改造、内环路路灯更新等工程。建成农村等级公路1608.5公里，等级公路通村率达到87%。市区空气环境质量明显改善，切实维护了群众环境权益。

　　统筹发展社会事业。全市共向国家、省申报各类科技项目37项。新启动建设标准化学校20所，改造校舍面积7.1万平方米。整合教育资源，合并中小学128所。高度重视甲型H1N1流感防控，防控救治工作取得了明显成效。围绕庆祝建国60周年开展了系列活动。我市闯关东年画等5项非物质文化遗产，被省政府列入省级保护名录。群众体育活动深入开展。统筹解决人口问题，人口自然增长率为3‰。全力打造特色旅游品牌，在评选“吉林八景”活动中，我市向海国家级自然保护区在全省42个参选景区、景点中脱颖而出，评为“吉林八景”第三位，被命名为“向海舞鹤”。

　　强化社会管理工作。加强信访工作，全面落实信访责任制。在国庆60周年期间，我市是全省唯一一个实现进京非正常上访和集体访“双零”目标的地级市。推进“平安白城”建设，加强治安整体防控体系建设，较为出色地完成了国庆60周年等大型活动安保任务。抓好“五五”普法，依法治市不断加强。注重行政复议工作，行政争议有效化解。完善食品药品监管机制，确保了人民群众饮食用药安全。突出“安全生产年”活动主题，全市安全生产形势保持基本平稳。国防动员和后备力量建设进一步加强。深入开展双拥活动，军政军民团结得到了巩固发展。圆满完成了“和平使命—2009”中俄联合反恐军事演习接待保障工作，受到中央军委总部和省委、省政府的表扬。对口援建四川地震灾区卡龙镇公路建设项目在全省率先完成。

　　（十）稳妥推进政府机构改革，加强服务型政府建设。认真组织实施政府机构改革，市政府工作部门由改革前的35个减为32个。协调推进县（市、区）政府机构改革，圆满地完成了机构改革任务。全市国有粮食购销企业产权制度改革按期完成。集体林权制度改革全面铺开。主动接受人大的法律监督、工作监督和政协的民主监督。办理人大代表建议、政协委员提案等104件，办复率均达到了100%，满意率分别为100%、98.6%。加强党风廉政建设，行政监察、审计监督深入推进。强化政行风和软环境建设，组织51个重点部门、重点行业在媒体进行公开承诺，政务环境得到了优化。加大车辆超限超载违法专项整治力度，治超工作取得了阶段性成果。组织开展了政府绩效评估工作。充分发挥市长公开电话作用。加强机构编制管理。扎实开展政府系统学习实践科学发展观活动，推动政府工作进一步改进。

　　一年来，民族宗教、人防、外事侨务、妇女儿童、老龄、气象、地方志和残疾人等工作都取得了新的成绩。

　　各位代表！

　　在应对国际金融危机严重冲击下，我们能够取得这样的成绩，确实来之不易。这是市委科学应对、正确领导的结果，是人大依法监督和政协民主监督、全力支持的结果，是全市200多万人民团结一心、共同奋斗的结果。在这里，我代表白城市人民政府，向为加快白城发展做出无私奉献、付出辛勤劳动的全市人民，致以崇高的敬意！向给予政府支持的人大代表、政协委员，向各民主党派、工商联、无党派人士和社会团体，向人民解放军驻白部队、武警官兵和公安司法干警，向中省直单位，向所有关心、支持白城发展的各界朋友，表示衷心的感谢！

　　在肯定成绩的同时，我们也清醒地认识到，全市经济社会发展和政府工作还存在着一些问题。一是农业遭受严重旱灾，农民增收压力加大，农村居民人均收入小幅下降。二是工业经济总量小，持续较快增长基础不牢固，部分行业、企业生产经营仍然十分困难。三是中小企业资金短缺问题还没有从根本上解决，制约了中小企业的发展。四是在国际金融危机严重冲击下，镇赉、大安两个县市受“油田经济”影响财政收入下滑，导致全市财政收支矛盾较为突出。五是群众关注的民生民利实事，个别完成的不够好，与群众期望还有差距。六是社会不稳定因素仍然存在，社会管理水平有待进一步提高，维护社会和谐稳定任务还很艰巨。

我们一定要增强发展信心和危机意识，在成绩面前保持冷静，在问题面前充满信心。以高度负责的精神，更加努力地做好政府工作，绝不辜负全市人民的期望和重托。

　　二、2010年工作总体安排

　　2010年，是实施“十一五”规划的最后一年。做好今年政府工作，对加快推进白城崛起振兴，为“十二五”规划启动实施奠定良好基础具有十分重要的意义。今年政府工作总的要求是：全面贯彻党的十七届四中全会、省委九届八次全会、全省经济工作会议和市委四届六次全会精神，以邓小平理论和“三个代表”重要思想为指导，深入贯彻落实科学发展观，抓住《国务院关于进一步实施东北地区等老工业基地振兴战略的若干意见》、《长吉图开发开放先导区规划》实施机遇，围绕建设吉林西部重要的“五大基地”、加快推进大能源、大园区、大通道、大生态、大环境、大民生“六大建设”，大力实施开发区“二次创业”，着力扩大经济总量，调整经济结构；着力抓好项目建设，扩大投资规模；着力完善基础设施，推进城镇建设；着力加强招商引资，强化开发开放；着力解决民生问题，促进社会和谐，努力开创我市经济社会发展的新局面。

　　2010年，全市经济和社会发展主要预期目标是：地区生产总值完成430亿元，同比增长19%；固定资产投资完成490亿元，同比增长40%；规模以上工业增加值完成59亿元，同比增长18%；地方级财政收入完成14.9亿元，同比增长18%;社会消费品零售总额完成164亿元，同比增长20.6%；城镇登记失业率控制在4.6%以内；城镇居民人均可支配收入15800元，同比增长6.8%；农村居民人均纯收入4000元，同比增长14.6%；人口自然增长率控制在4‰以内。

　　以上这些主要指标，是经过综合权衡各方面因素，按照增幅力争高于全省平均水平、高于自己同期水平、高于有可比性的省内兄弟城市和周边地区水平“三个高于”的原则确定的。没有一个较高速度的发展，我市与省内较大城市发展距离会越拉越大，与全省的发展步伐会显得更加不协调。目前，我们是围绕增加经济总量，保增长、保速度的发展，既要增强社会各方面的发展信心，又要保证经过努力能够实现，促进全市经济社会发展迈上新台阶。

　　今年，重点要抓好以下十个方面工作：

　　（一）积极扩大投资规模，集中力量抓好重大项目建设

　　坚持投资拉动、项目带动。全市规划实施3000万元以上重点项目170个，总投资1302亿元，年度完成投资393.8亿元。其中，亿元以上项目达到115个，10亿元以上项目达到24个。固定资产投资增幅要力争高出全省11个百分点。加快项目建设步伐，坚持前期项目抓推进、新建项目抓开工、在建项目抓投产，形成大干、快上的局面。

　　积极向上争取投资项目。抓紧编制“十二五”规划，争取重大项目、重点项目纳入国家和省布局。把握国家和省投资导向，紧紧围绕重点支持领域，做好向上争取工作，努力争取国家和省最大的扶持。积极开展“赴省进京”工作，各县（市、区）、市直各部门都要行动起来，抓住国家和省政策的支持点，找准向上争取的切入点，同省和国家有关部门搞好对接，力求向上争取投资项目实现新的突破。

　　抓好重大项目谋划储备。抓住国家增加对“三农”、科技、教育、卫生、文化、社会保障、保障性住房、节能环保等方面和中小企业、居民消费、欠发达地区支持力度，以及加快发展新能源、新材料、新医药等战略性新兴产业的政策机遇，超前谋划一批带动作用强、辐射范围大、发展后劲足，能够立市立县或国家级重大项目，特别是10亿元以上的大项目，做到建设一批、论证一批、储备一批，为增强发展后劲奠定良好基础。

　　（二）抓住产业跃升计划实施契机，培育优势支柱特色产业

　　发展壮大优势支柱产业。围绕国家发展战略性新兴产业，抓住省委、省政府实施八大产业跃升计划契机，做大做强支柱优势特色产业，大力发展新能源产业，培育壮大能源这个第一支柱产业。做好千万千瓦级风电基地、千万千瓦级火电基地以及油气、光热资源开发等规划编制申报工作，力争纳入省和国家“十二五”规划。抓好洮南向阳、通榆瞻榆、洮北宝山等风电场开发，力争新增并网发电100万千瓦以上。加快推进中电投白城发电公司二期工程2×100万千瓦项目前期工作，争取在“十二五”初期启动建设。搞好大安大唐、镇赉华能火电项目推进工作。发展热电联产项目，加快白城热电厂供热改造项目建设。加大光伏开发力度，促进通榆、洮南、镇赉、大安太阳能发电项目建设。注重引进推进生物质能项目建设。建成通榆500千伏输变电工程。协调争取洮南、大安、镇赉输变电工程项目。强化油气开发项目建设，争取油气产量达到180万吨以上。做优做强汽车零部件、纺织服装、医药、建材等传统优势产业。

　　突出打造龙头强势企业。坚持以增量带存量，围绕培育一批产值超10亿元、30亿元、50亿元大企业，带动产业跃升、促进结构调整。扶持壮大白城德尔福派克电气有限公司，培育发展白城金升镍业有限公司，促进黑龙江瑞好集团白城工业园风电有限公司达产达效。加快投资120亿元的华锐风力发电设备制造、投资100亿元的通榆三一风电产业园、一期投资2.5亿元的西班牙歌美飒风机制造等项目建设。强化小企业成长工程建设，力争全年规模以上工业企业突破300户。注重盘活存量，加快企业招商重组步伐，加大现有企业扶持力度。深入开展全民创业活动，促进民营经济大发展。

　　加快推进工业技术改造。抓住国家支持新一轮老工业基地技术改造有利时机，实施好重点企业技术改造三年滚动计划。今年，要重点抓好20个投资5000万元以上的技改项目。强化节能减排和环境保护工作，加快推进我市纳入《松花江水污染防治规划》12个项目建设。坚持实施品牌战略，培育企业品牌和名牌产品，抓好白城中一精锻等产品申报国家驰名商标工作，充分发挥品牌效应。

　　（三）加强粮食生产基地建设，提高粮食综合生产能力

　　加快水利工程项目建设。完成引嫩入白、大安灌区主体工程，推进洮儿河灌区、月亮湖蓄滞洪区、引洮分洪入向、中小河流域治理等配套建设。抓紧完成吉林西部土地开发整理镇赉、大安项目区建设。积极向上争取节水井灌项目，力争打抗旱水源井3000眼，新增节水灌溉面积50万亩，使旱田全程灌溉面积达到500万亩，增强抗旱能力。

　　强化现代农业园区建设。加快建设镇赉、大安省级生态农业示范区，推进13个重点项目建设。抓好国家整县推进现代烟草农业试点，建设国家级现代烟叶示范区。发展棚模和庭院经济，做大集中连片棚模经济发展区。启动建设各类农业科技园区32个，推进农业集约化、规模化、产业化发展。继续实施200万亩盐碱地脱硫废渣改造项目。落实畜牧业加快发展三年攻坚计划，突出抓好45万头肉牛产业园区、35万吨乳品产业园区建设，带动养殖规模扩大、效益提高，建设牧业强市。

　　推进农业产业化经营。加快培育壮大产业化龙头企业，启动建设40个超千万元农产品加工项目，建设5个产地专业加工园区。推进益海嘉里粮油综合加工等一批超亿元龙头企业建成投产。加强产业化龙头企业基地建设，为构建吉林西部特色农产品加工基地奠定基础。

　　（四）加快开发区“二次创业”步伐，充分发挥引领带动作用

　　科学进行定位。坚持以特色立区，白城工业园区要重点发展工业项目，加快建成白城工业主体区；白城经济开发区、民营经济开发区要在发展工业项目的同时，重点发展商贸流通项目，加快建成区域商贸核心区；查干浩特旅游经济开发区要重点发展旅游项目，加快建成具有白城生态和民俗特色的旅游区；大安经济开发区要统筹抓好工业项目和旅游项目，加快建成工业和旅游新区。各县（市）工业集中区要按照差异化、配套化、特色化的原则，确定功能定位，促进重点产业集聚发展。

　　壮大经济总量。坚持把项目建设作为扩总量、增实力的主要抓手，做到一手抓引进，组织开展招商引资；一手抓推进，加快项目建设进度。各开发区招商引资额度、主要经济指标都要争取比去年翻一番。千方百计加大项目引进力度，壮大开发区实力。对已引进项目要加快建设速度，尽快投产达产，使开发区壮大起来、发展起来。

　　创新体制机制。坚持扩权强区，赋予开发区更多的经济管理权限，建立新型管理体制。开发区要更多的强化招商、服务职能，干部人事管理应当更加灵活而有效率，力求“办事不到市”、“办事不离区”，实行封闭管理，使开发区建设成为自主创业区。

　　（五）开展“举市三年大造林”活动，推进荒漠化治理进程

　　明确目标任务。从2010年到2012年末，利用三年时间，全市完成植树造林100万亩，基本形成城市园林化、农田林网化、村屯林围化、庭院林果化、道路林荫化、湿地景观化的建设格局。

　　实施绿化工程。围绕“举市三年大造林”目标任务，组织实施城市绿化美化、乡村绿化美化、绿色通道、农田防护林、荒山荒地湿地治理五大工程建设。城市绿化覆盖率达到38%以上，村屯绿化覆盖率达到30%以上，使“举市三年大造林”活动起好步、开好局。

　　争取扶持政策。抓紧协调编制吉林西部荒漠化治理项目规划，力争纳入国家和省生态建设布局。全力争取三北四期、荒山荒地造林、西部治碱、生态草等国家和省项目资金。充分利用省林业厅给予的20条优惠政策，打好“举市三年大造林”攻坚战。推进集体林权制度改革，确保全市在今年春耕前完成集体林权制度改革任务。全面完成“十一五”防沙治沙任务，使生态产业建设取得阶段性、突破性成果。

　　（六）发挥区域商品集散地功能，做大做强现代服务业

　　加强对外通道建设。推进长白快速铁路、白城站扩能改造、白阿铁路线增建复线等项目建设。加强市区金辉公铁立交桥，以及镇赉、洮南公铁立交桥项目建设。力保珲乌高速公路白城段建成通车，开工建设嫩通高速公路、白城民航机场项目。铁路、公路、航空三大通道建成后，我市将在全省率先实现县县通高速公路，成为继长春之后高速公路环城的地级城市，形成内蒙古东部、黑龙江南部和吉林西部三省区交通枢纽和物流中心。

　　全力打造物流平台。抓好白城内陆港物流中心等项目建设，加快建成粮食、建材等大宗商品集散中心城市。整合扩建杂粮杂豆、水产品、畜产品等有形市场。培育壮大白城欧亚购物中心，带动连锁、代理等新型流通业态发展。加快“万村千乡市场工程”、“双百市场工程”和“新网工程”等建设，促进商业网点向农村延伸。重点抓好家电、汽车摩托车下乡工作，全面落实家电和汽车以旧换新政策，充分发挥消费拉动经济增长作用。

　　加快做大旅游产业。围绕贵在原始、美在自然，加大以向海、莫莫格自然保护区为代表的重点景区、景点推介力度，加强草原、湿地、风电、嫩江等旅游资源开发项目建设。搞好向海湿地生态示范区建设、莫莫格湿地恢复、嫩江湾湿地开发、安白百公里新能源综合景观带建设等项目，促进旅游产业加快发展。

　　（七）抢抓长吉图规划实施有利机遇，加大开发开放力度

　　抢抓开发开放机遇。抓住全省启动实施经国务院批准的长吉图开发开放先导区规划，充分利用我市作为联合国开发计划署规划确定的第四条欧亚大陆桥重要节点城市这一优势，积极融入长吉图开发开放先导区建设，加快构建“五大基地”、推进“六大建设”，强化产业链条衔接、生产要素互补等，在政策创新、金融创新、对外开放上实现新的突破，促进全市大开发、大建设、大发展。

　　深入开展对接活动。利用长吉图开发开放先导区合作平台，发挥我市地处吉林、黑龙江、内蒙古三省区交界处的比较优势，谋划一批旅游、物流、交通、能源、科技和生态环保等方面跨省区合作的大项目。特别是要进一步加强与蒙古国和蒙东地区的跨省通道建设、能源建设等区域对接合作项目，促进区域联动开发、共同发展。

　　加大招商引资力度。积极“走出去、请进来”，进一步扩大对外交流与合作，组织好赴“环渤海”、“长三角”、“珠三角”等地招商引资活动，开展好鲁商、粤商、浙商和川渝企业家白城行活动。实行点对点、一对一招商，由重点一线城市招商向一、二线城市并重转变，不断拓宽招商引资领域。抓住今年国家“两会”契机，开展好与央企、民企对接活动，在合资合作上实现新的突破。全年引进资金要达到328亿元，同比增长30%，拓宽对外开放广度和深度。

　　做实做大融资平台。充分发挥“一主四辅”融资平台作用，积极与省内金融机构及各类投资机构合作。创新中小企业贷款方式，探索以产权、市场、资产、技术等资源置换资金。实施“企业上市培育工程”，积极谋划推进白城中一精锻等骨干企业上市融资。建立政、银、企“零距离”对接机制。各级金融机构要走出怕风险、多吸储、少放贷的误区，用好用活适度宽松的信贷政策，为企业提供高效的融资服务。企业要加强同各类金融机构的沟通协调，提高信贷信用度，促进银政银企合作实现大的突破。

　　（八）打好“城市建设管理年”攻坚战，努力改善城市环境

　　实施基础设施改建工程。今年市区基础设施要进行大投入、大改造、大建设，实施十个方面工程，新建1处市民休闲广场、维修和新建9条道路、改造二环路内7条主要街路人行道和边石、建设敞开式海明路商业区、完善和新建环卫设施、维修污水泵站、更新路灯设施、改造雨水管网、修建景点式污水雨水排放地、开展全面绿化等，使市区基础设施建设打一个翻身仗，市容市貌实现较大的改观。

　　抓好“双城创建”活动。组织开展卫生城、园林城“双城创建”活动。按照国家和省级卫生城创建目标，加强市容环境卫生整治、卫生监督和除害防病等重点工作；结合开展“举市三年大造林”活动，搞好城市园林规划和相关配套工程建设，提高城市绿化美化整体水平，努力实现省级卫生城和园林城工作目标。

　　（九）积极推进城镇化进程，加快社会主义新农村建设步伐

　　加快启动“百镇建设工程”试点镇建设。抓住省委、省政府实施“百镇建设工程”机遇，加强11个省级试点镇、9个市级试点镇建设，着力培育和发展主导产业，增强小城镇的吸纳人口和产业支撑功能。要统筹推进市、县城市和小城镇协调发展，做大做强市、县城市，带动小城镇发展。围绕突出特色，打造一批工业大镇、商贸大镇、旅游大镇，力争用5到7年的时间，使小城镇发展成为小城市。抓紧完成试点镇小城镇建设规划编制工作，按照“一镇一貌、一镇一品”的原则，今年力争在加强基础设施建设、改善环境、培育发展产业等方面取得重大进展。

　　加强“千村示范、万村提升”工程建设。全面贯彻落实全省社会主义新农村建设现场会暨启动“千村示范、万村提升”工程动员大会精神，根据省、市、县、乡四级新农村建设试点村、推进村，按照区位优势、经济基础、基础设施、发展潜力等方面条件，确定示范村、提升村，研究制定示范村、提升村经济社会发展总体规划、建设规划和村屯改造方案，明确建设项目、重点任务和分段推进目标，启动建设示范村、提升村工程，开创我市新农村建设的新局面。

　　（十）着力解决民生民利问题，全力维护好社会和谐稳定

　　办好民生民利实事。围绕保障和改善民生，努力解决民生民利问题。一是千方百计促进创业就业。深入推进全民创业促就业工作，增加就业扶持资金和小额担保贷款，将小额担保贷款基金增至4000万元，累计发放小额担保贷款达到1.6亿元。积极推进高校毕业生创业园建设。新增城镇就业3.7万人，转移农村劳动力34万人。开发公益性岗位保持在1.1万人就业规模。保持城镇零就业家庭动态为零。二是加快完善社会保障体系。搞好新型农村养老保险制度试点。新型农村合作医疗参合率，力争在现有的基础上再提高1个百分点。城镇居民基本医疗保险参保人数保持47.4万人。城镇职工基本医疗保险参保人数保持33.5万人。完善城乡低保制度。建立健全城乡医疗救助制度。三是改善城乡居民居住条件。改造城市棚户区及危旧房100万平方米，建设城市廉租住房10万平方米。改造农村泥草房5.6万户。四是加强乡村交通设施建设。投资6.8亿元新改建农村公路932.9公里，建设三级以上客运站3个、五级乡村客运站22个、货运站1个。五是改造供热供水管网。加强城市热源能力建设，保障居民冬季供热，解决好“暖房子”问题。改造市区供热管网8公里，集中供热普及率达到83%；供水管网3公里，供水普及率达到90.2%。六是实施天然气城市气化工程。积极引进资金，争取实施天然气入户工程。七是强化医疗卫生设施建设。推进白城中心医院易地新建、白城中医院新建、白城第三人民医院病房楼改造项目建设。加强农村三级卫生服务网络和新型城市卫生服务体系建设。八是实施扶贫开发推进计划。确保完成省下达的脱贫人口任务、农村人口饮水安全工程建设任务。九是改善农村义务教育办学条件。继续实施“新农村百校标准化建设工程”，新建和改造标准化学校15所。加快推进中小学校校舍安全工程建设。全面完成农村初中物理、化学、生物实验室和微机室建设。十是加大公益性文化事业投入。建设12个乡（镇）综合文化站、100个农村文化大院、500个农家书屋。加强白城电影城项目建设，组建农村放映队，丰富广大人民群众文化生活。

　　加快社会事业发展。实施科技发展计划，全力争取国家和省科技项目，培育科技型创新企业。优化教育资源配置，健全义务教育经费保障机制。推进医疗卫生体制改革，完善公共卫生疾病防控体系和医疗救治体系，继续做好甲型H1N1流感等新型传染病防控救治工作。搞好文化体制改革，加快文化产业发展。深入挖掘人文历史资源，加大宣传和推介力度，打造白城软实力。组织开展全民健身活动。抓好人口和计划生育工作，稳定低生育水平，统筹解决人口问题。加强和改进人才工作，积极吸引我市急需的各类人才。注重国防动员和后备力量建设。坚持以新一轮双拥模范城命名为契机，深入开展双拥工作。保障妇女儿童权益，发展老龄和残疾人事业，做好民族宗教、气象、人防、地震、外事侨务、地方志等工作。

高度重视社会管理。深入开展“平安白城”建设，强化社会治安防控体系建设，加大打击刑事犯罪力度，坚决防止重特大恶性事件发生。推进“五五”普法，深化法制宣传教育和法律服务，做好人民调解工作。加强信访工作，严格落实领导责任制和责任追究制，坚持依法规范信访秩序，切实维护群众信访权益。深入推进安全生产执法、治理、教育“三项活动”，加大对安全生产隐患排查和整治力度，有效防范和坚决遏制重特大事故的发生，促进全市安全生产形势持续稳定好转。注重应急管理工作，科学预防和妥善处置各类突发事件。加强食品药品安全监管，确保群众饮食、用药安全，全力维护广大人民群众利益。

　　三、加强政府自身建设

　　应对复杂多变的经济形势、面对艰巨繁重的发展任务，政府工作面临着严峻的挑战和考验。要求我们积极推进政府管理创新，加强政府自身建设，为促进全市经济社会又好又快发展提供坚强保障。

　　（一）加快转变政府职能。巩固政府机构改革成果，进一步理顺部门职责关系，优化政府组织结构，切实推进政企分开、政资分开、政事分开和政府与市场中介组织分开。坚决取消和下放政府不该管的事项，有效解决政府运行中权责脱节、重权轻责、缺乏监督的问题。建立健全科学民主决策机制，重大决策的形成和执行都要加强调查研究，做到察民情、听民意、聚民智，尊重客观规律，提高决策的预见性、科学性和有效性。积极推进政府信息公开，完善发布机制，增强透明度，保障人民群众的知情权、参与权、表达权、监督权，让人民群众知道政府在想什么、做什么，使政府工作赢得人民群众的充分理解、广泛支持和积极参与。

　　（二）努力提高行政效能。坚持依法行政，深入推进行政审批权相对集中改革，加强和改进政务大厅工作，全力打造阳光工程。实行软环境建设目标管理责任制，深入开展软环境和政行风评议活动，做好项目服务和投资者权益保障工作，创造良好的发展环境。突出解决不依法办事、不执行政策、推诿扯皮甚至刁难勒索等问题。自觉接受人大法律监督和政协民主监督，积极主动做好人大代表建议和政协委员提案办理工作。充分发挥监察、审计监督作用，高度重视人民群众监督和新闻舆论监督。抓好行政复议、行政诉讼等工作，促进行政效能提高，以实际行动取信于民。

　　（三）解放思想转变政风。坚持在解放思想中谋发展、抓发展、促发展，凡是有利于加快白城发展的事情，就要大胆地试、大胆地闯、大胆地干，绝不允许因思想不解放贻误机遇、影响发展。围绕提高政府执行力，加强政风建设，全面开展政府绩效评估，实行行政问责制。深入开展“发展杯”竞赛活动，进一步完善调度、通报、奖惩制度。采取综合治税措施，加大税收征管和非税收入力度，从严控制一般性支出。强化增收节支，严格资金管理。严格人事编制管理。办好市长公开电话，为群众解难题、办实事、谋福祉。抓好重点领域改革。坚持从严治政、廉洁从政，构建惩防并举的反腐倡廉工作机制。进一步推进政府制度建设，做到用制度管权、用制度管事、用制度管人，强化对专项资金、重大投资项目和权力部门的审计和监察。坚决纠正损害群众利益的不正之风，建设为民、务实、廉洁、高效的人民政府。

　　各位代表！

　　我们正处在一个新的历史征程上，面临的任务艰巨而繁重，肩负的责任重大而光荣。让我们在市委的领导下，在市人大监督和政协民主监督下，深入贯彻落实科学发展观，解放思想，开拓奋进，埋头苦干，务求实效，为加快白城崛起振兴创造出新的业绩，绝不辜负全市人民的信任和期待！
